# Malignant ascites in ovarian cancer: New advances and translational opportunities

**DOI:** 10.1016/j.tranon.2025.102568

**Published:** 2025-10-16

**Authors:** Kyung Hyun Boo, Gaeun Lee, Minkyung Song

**Affiliations:** a Department of Integrative Biotechnology, Sungkyunkwan University, Suwon, Republic of Korea; b Department of Biopharmaceutical Convergence, Sungkyunkwan University, Suwon, Republic of Korea

**Keywords:** Malignant ascites, Ovarian cancer, TME reprogramming, Immune suppression, Therapy resistance

## Abstract

Malignant ascites is a defining and clinically challenging feature of advanced ovarian cancer, driving peritoneal dissemination, therapeutic resistance, and poor clinical outcomes. Beyond its traditional description as a protein-rich fluid, high-resolution profiling now reveals ascites as an active ecosystem that enforces vascular leakage, creates dynamic interactions among cellular, acellular, and environmental components, fuels cellular reprograms, and builds profound immune suppression. In this review, we first synthesize established knowledge on ascites formation and composition, and then delineate how this milieu reshapes tumor, stromal, and immune compartments, altering their fate and function. We also integrate current diagnostics and map the therapeutic landscape spanning vascular permeability control, effective locoregional chemotherapy, cellular reprogramming, and stress/metabolic interventions, alongside palliative drainage devices. A deeper understanding of the dynamic interactions and regulatory mechanisms within malignant ascites provides a foundation for developing new therapeutic strategies to overcome its clinical challenges and improve outcomes for ovarian cancer patients.

## Highlights

• Malignant ascites is an active ecosystem that drives peritoneal spread, therapy resistance, and immune evasion in advanced ovarian cancer.
• Malignant ascites reprograms tumor, stromal, and immune compartments and stress-metabolic coupling is emerging mechanistic core.
• Deeper biologic insight is clinically actionable, enabling ascites-specific biomarkers and risk stratification to overcome current treatment limitations.
• Therapeutic opportunities are expanding and biomarker-enriched trials with patient-centered endpoints to convert mechanism into durable benefit.

## Graphical abstract

**Figure:**
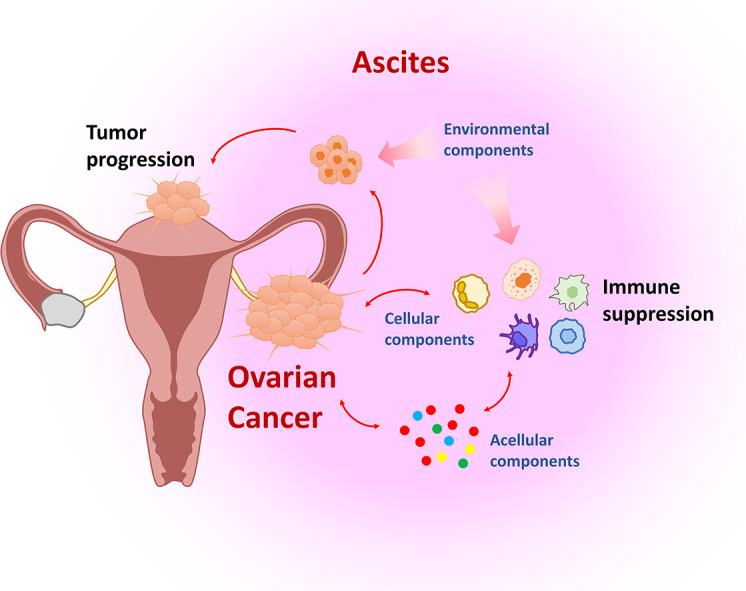

## Introduction

Ovarian cancer is the most lethal gynecological malignancy worldwide, with most cases diagnosed at advanced stages (FIGO stages III/IV) [1]. The asymptomatic nature of early-stage disease significantly hampers early detection; by the time symptoms appear, metastasis is common, resulting in a poor prognosis. Standard management−optimal cytoreductive surgery followed by platinum-taxane chemotherapy−often induces an initial remission, yet most patients ultimately relapse as chemoresistance emerges [2]. Consequently, five-year survival remains dismal, at approximately 30 % for advanced-stage disease [3].

One of the hallmarks of advanced and relapsed ovarian cancer is malignant ascites, the pathological accumulation of peritoneal fluid [1]. Ascites is closely linked to disease stage and burden: it is substantially more prevalent and voluminous in advanced diseases than in early-stage diseases, and average fluid volume rises with increasing peritoneal tumor number and size [1]. Ascites causes abdominal distension, early satiety, and dyspnea, and patients frequently require repeated paracenteses, which carry their own risks, such as pain, bleeding, visceral injury, and infection [1]. Clinically, the presence of ascites correlates with suboptimal cytoreduction and reduced sensitivity to systemic therapy, indicating that it is more than a bystander and may actively impede treatment efficacy [4]. Qualitatively, hemorrhagic ascites, characterized by elevated red blood cell counts, is associated with a worse prognosis than non-hemorrhagic fluid [1].

More recently, ascites has been profiled at single-cell and molecular resolution. Single-cell RNA sequencing and multiparameter flow cytometry delineate the lineage composition and functional states of tumor, stromal, and immune cells within the fluid, capturing plasticity and heterogeneity that bulk measures obscure [5,6]. Complementary multi-omics−including transcriptome, proteome, metabolome, and lipidome−resolve metabolic pressures and stress programs at scale, while liquid biopsy approaches applied to ascites−leveraging cell-free nucleic acids and vesicle-associated cargo−enable longitudinal, minimally invasive monitoring [[7], [8], [9], [10], [11], [12]]. Together, these high-resolution readouts can identify mechanisms of therapeutic resistance emerging in the peritoneal ecosystem, nominate actionable targets in cellular, acellular, or environmental nodes, and support patient stratification and real-time response assessment. Such data creates an opportunity to couple current fluid management with disease-modifying strategies that also consider how ascites shapes tumor behavior and immune landscape.

Accordingly, this review first condenses the essential background on the formation and composition of malignant ascites; second, contextualizes recent findings showing how ascites conditions tumor/stromal compartments and impairs anti-tumor immunity; and lastly translates these insights into detection/diagnostic frameworks and therapeutic avenues relevant to ascites-predominant disease. We aim to provide a balanced guide to recent literature and a practical roadmap for leveraging profiling to inform mechanism-based interventions.

## Formation and composition of ovarian cancer ascites

**Fig. 1:**
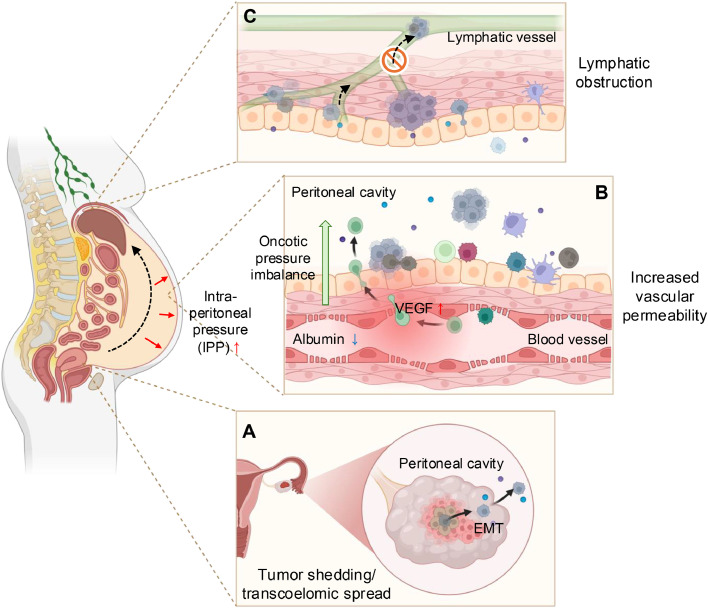
Formation of malignant ascites in ovarian cancer. Malignant ascites results from three cooperating processes−tumor dissemination, increased vascular permeability, and impaired lymphatic drainage. (A) Tumor shedding/transcoelomic spread: ovarian tumor cells undergo epithelial–mesenchymal transition (EMT), detach into the peritoneal cavity, and form multicellular spheroids that survive in the peritoneal fluid and seed mesothelial surfaces. (B) Vascular permeability and oncotic imbalance: tumor- and stroma-derived mediators, exemplified by VEGF, increase vascular permeability; albumin and plasma proteins extravasate, disrupting the plasma−peritoneal oncotic gradient and driving ultrafiltrate into the peritoneal cavity. An influx of stromal/immune cells accompanies fluid accumulation. (C) Impaired lymphatic drainage: tumor implants and inflammatory remodeling obstruct diaphragmatic/omental lymphatic vessels, impeding peritoneal fluid clearance. Collectively, these processes cause progressive ascites buildup and contribute to elevated intraperitoneal pressure (IPP). This image was created with BioRender.com.

Under physiological conditions, small volumes of peritoneal fluid lubricate the serosal surfaces and are cleared predominantly through diaphragmatic lymphatics, maintaining neutral net balance [13]. In ovarian cancer, this equilibrium is disrupted by transcoelomic dissemination: tumor cells shed from primary sites, aggregate into free-floating spheroids, and adhere to mesothelial extracellular matrix to establish secondary implants [14] (Fig. 1). These implants, mesothelial injury and inflammatory remodeling impair lymphatic drainage, while tumor-derived mediators (e.g., VEGF, IL-6, IL-8) increase the microvascular permeability and albumin leak into the peritoneal cavity [15]. Loss of the plasma-peritoneal oncotic gradient, combined with heightened filtration and reduced lymphatic clearance, drives net fluid accumulation [13]. The resulting ascites is typically protein-rich, low-serum-ascites albumin gradient (SAAG) exudate, distinct from portal-hypertensive transudates [16] (Table 1).

**Table 1:** Malignant versus non-malignant ascites: a comparative summary.

Diagnostic factor	Malignant ascites	Non-malignant ascites		
	Cancer	Portal hypertension	Infectious	Pancreatic
**Gross appearance**	Milky† or bloody	Milky	Various (milky, cloudy, turbid)	Various (milky, cloudy, turbid)
**Protein concentration**	Exudate (≥2.5 g/dL)	Transudate (<2.5 g/dL)	Exudate (≥2.5 g/dL)	Exudate (≥2.5 g/dL)
**SAAG**	Low (<1.1 g/dL)	High (≥1.1 g/dL)	Low (<1.1 g/dL)	Low (<1.1 g/dL)
**LDH activity**	Increased	Decreased	Increased	Increased
**Glucose level**	Decreased	Serum level	Decreased	Decreased
**Tumor markers**	CA125, HE4, CEA, CA19–9	Rarely observed in some conditions		
**VEGF level**	Increased	Decreased		

† Milky^#^ suggests chylous ascites (often triglycerides ≥ 200 mg/dL). Overlap may occur−malignant ascites can show a high SAAG when portal hypertension coexists; laboratory results must be interpreted with clinical context and other examination. *Abbreviations:* SAAG, serum-ascites albumin gradient; LDH, lactate dehydrogenase; VEGF, vascular endothelial growth factor; CEA, carcinoembryonic antigen; CA19–9, carbohydrate antigen 19–9; HE4, human epididymis protein 4.

**Fig. 2:**
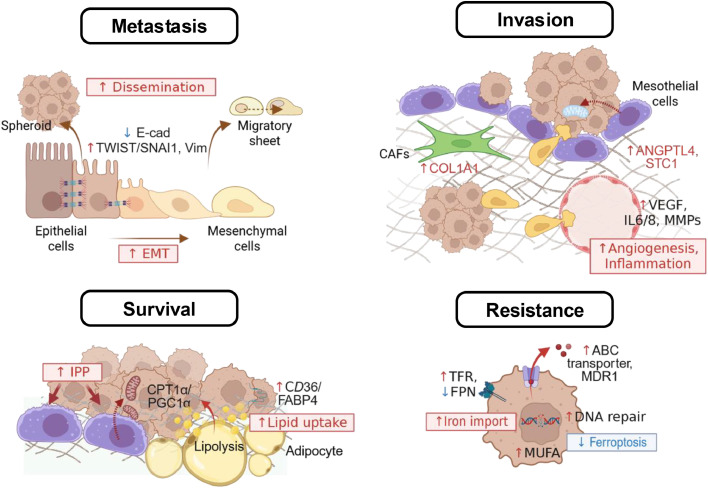
Tumor and stromal reprogramming by malignant ascites. Components of malignant ascites remodel cancer and stromal cells, promoting metastasis, invasion, survival, and treatment resistance. Ascites pushes epithelial cells into unstable epithelial–mesenchymal intermediate states, enabling collective migration as spheroids or migratory sheets. Ascites stimulates fibroblasts and reprograms mesothelial cells, fostering mesothelial transition, glycolysis, and motility. VEGF and inflammatory cues increase endothelial/mesothelial permeability and angiogenesis, facilitating trans-mesothelial invasion. Lipid-rich ascites and omental adipocyte lipolysis fuel tumor growth. Tumors increase fatty acid uptake and enhance mitochondrial activity, sustaining OXPHOS and membrane/redox homeostasis. IPP elevation further supports peritoneal survival. Chronic exposure induces drug-efflux programs, rewires iron/lipid metabolism, augments DNA repair pathways, protects from ferroptosis, collectively promoting resistance. Abbreviations: ANGPTL4, angiopoietin-like 4; STC1, stanniocalcin-1; COL1A1, collagen type I alpha 1 chain; MMP, matrix metalloproteinase; IPP, intraperitoneal pressure; CPT1α, carnitine palmitoyl transferase 1α; PGC1α, peroxisome proliferator-activated receptor-γ coactivator-1α; FABP4, fatty-acid–binding protein 4; ABC, ATP-binding cassette; MDR1, multidrug resistance protein-1; TFR, Transferrin receptor; FPN, ferroportin; MUFA, monounsaturated fatty acid. This image was created with BioRender.com.

Ovarian cancer ascites is highly heterogeneous; its cellular, acellular, and environmental composition has been extensively characterized [1,13,17]. Recent single-cell RNA-seq datasets−GSE146026 [5] and PRJCA005422 [6]−now provide a higher-resolution landscape of ascites, resolving functionally distinct malignant, stromal, and immune states (Fig. 2). In malignant epithelial cells, scRNA-seq delineates inter-patient heterogeneity driven by copy-number alterations and stemness programs, while also revealing IL-6-responsive inflammatory modules mirrored in cancer-associated fibroblast (CAFs), nominating JAK-STAT as a shared vulnerability [5]. The stromal niche is composed of immunomodulatory CAFs, mesothelial cells, and endothelial cells [5]. CAFs secrete IL-6, CXCL12, complement factors and remodel collagen networks, which in turn activate JAK-STAT and NF-kB programs in neighboring cancer cells; DES^+^ mesothelial cells act as pro-metastatic and immunoregulatory partners by recruiting T cells and macrophages via the CXCL12–CXCR4 pathway, while also scaffolding adhesion and remodeling the peritoneal niche; and endothelial profiling further identifies 13RΑ1^+^ endothelial cells whose abundance correlates with platinum-based chemotherapy response, underscoring how vascular programs integrate with ascites-derived cues [5,6,18].

In parallel, single cell lineage and trajectory analyses refine the immune landscape [6]. Ascites-enriched macrophages upregulate S100A8/A9 and downregulate HLA-II genes, displaying developmental origins distinct from tumor-resident counterparts and acquiring dysfunctional, tumor-promoting phenotypes [6]. Dendritic cell subsets show site- and response-linked programs, with cDC1 (CLEC9A^+^) and cDC2 (CD1C^+^) enriched in ascites; notably, platinum-nonresponsive patients exhibit increased cDC2 and reduced cDC1 [6]. Among T cells, a GZMK^+^ effector-memory CD8^+^ subset resides between naïve/central-memory and exhausted states and transitions toward intratumoral exhausted T cells, suggesting that ascites serves as a feeder pool [6]. Single cell transcriptomic profiling further identifies mucosal-associated invariant T (MAIT) cells within ascites, with their relative abundance proposed as a potential predictive marker for chemotherapy response [6].

Recent proteome- and metabolome-oriented studies demonstrate that the ascites supernatant captures dynamic metabolic states of ovarian cancer during progression, linking soluble factors to pathway activation in both cancer and host cells [10]. The soluble mediators−VEGF, EGF, IL-6/IL-8/IL-10 and complement factors−promote vascular leak, inflammation, and immune suppression [1]. Extracellular vesicles (EVs) carry a transferable cargo of miRNAs, proteins, and lipids that reprogram recipient cells and serve as accessible analytes for liquid biopsy [7,8]. Metabolites and lipids (e.g., lactate, acetate, cholesterol species, LPA) reflect active metabolic remodeling and provide both fuel and signaling cues [8,19,20]. Iron-handling proteins (e.g., transferrin, ferritin) and other stress-related cargos are frequently detected and may correlate with therapy tolerance [21,22].

Beyond composition, malignant ascites imposes distinctive biochemical and biomechanical constraints that shape cell behavior. Relative hypoxia, nutrient competition, and alkaline pH (often >7.5), conditions that favor spheroid viability and alter immune cell metabolism [[23], [24], [25]]. Progressive fluid accumulation raises intraperitoneal pressure (IPP) and generates fluid flow and shear forces with respiration and peristalsis; these mechanical forces promote tumor cell shedding and redistribution, and have been linked to spheroid formation, cytoskeletal remodeling, and treatment resistance [26,27]. Collectively, these features define a permissive peritoneal niche that sustains tumor growth and undermines therapeutic efficacy.

## Tumor and stromal reprogramming by malignant ascites

Malignant ascites reprograms cancer and stromal cells to promote peritoneal survival, invasion, metastasis, and treatment resistance. Phenotypically, exposure to ascitic fluid drives ovarian cancer cells into unstable epithelial-mesenchymal intermediate states, characterized by TWIST/SNAI1 induction, partial loss of E-cadherin, and vimentin gain [28]. These changes support collective migration while maintaining focal adhesion and pro-survival signaling [28]. Depending on their baseline epithelial-mesenchymal traits, cells form either spheroids or migratory sheets: more epithelial programs favor compact spheroids, whereas more mesenchymal programs favor migratory sheets [29]. Both trajectories are reinforced by αv integrins, which link fibronectin-rich matrices to PI3K-AKT-FAK signaling, thereby enhancing adhesion and motility [29]. Consistently, patient-derived ascites frequently yield hybrid epithelial-mesenchymal aggregates co-expressing PAX8/EpCAM with α-SMA, while ascites exposure upregulates fibronectin and α-SMA to strengthen the three-dimensional architecture required for peritoneal colonization [30]. The resulting spheroids often exhibit therapy-tolerant, stem cell-like features (e.g., CD24, CD44, CD117, CD133, MMP-9, EpCAM), along with increased expression of ABC drug transporters (e.g., ABCB1, ABCG2) that contribute to platinum and taxane resistance in advanced-stage disease [31,32].

Quantitative proteomic analyses have identified elevated levels of COL1A1, primarily produced by fibroblasts, along with other fibrillar collagens in ascites [18]. Malignant ascites stimulates fibroblasts to secrete COL1A1, which binds integrin β1 on cancer cells, activating AKT phosphorylation, increasing vascular permeability, and promoting cancer cell invasion and trans-mesothelial migration [18]. Ovarian cancer ascites reprograms mesothelial cells to secrete ANGPTL4 and STC1, which drive mesothelial- to-mesenchymal transition, glycolysis, and migration [33]. These cancer-associated mesothelial cells, in turn, enhance ovarian cancer cell adhesion, invasion, and proliferation, while also stimulating monocyte migration and endothelial tube formation [33]. In addition, VEGF in ascites increases peritoneal endothelial permeability by downregulating claudin-5 and upregulating MMP expression [34,35].

Malignant ascites also exhibits elevated levels of lipid derivatives, indicating both active fatty acid synthesis and catabolism [8]. Lipid-rich ascites−shaped by adipocyte lipolysis in the omentum and by tumor- and stroma-derived secretomes−feeds back to promote ovarian cancer aggressiveness [19]. Adipocytes and preadipocytes provide fatty acids and remodel the extracellular matrix to facilitate implantation; the CD36/FABP4 axis at adipocyte-tumor interfaces enhances fatty acid uptake and peritoneal metastasis [19]. Under glucose-deprived conditions, ovarian cancer cells rely on CPT1α-mediated fatty acid oxidation and PGC1α-driven mitochondrial programs, thereby sustaining oxidative phosphorylation, membrane biogenesis, and redox homeostasis; they also reprogram lipid metabolism toward *de novo* fatty acids synthesis: FASN activation drives PI3K-mTOR survival signaling [20].

Beyond fatty acid metabolism, specific lipid mediators in ascites further potentiate tumor progression. Lysophosphatidic acid (LPA), abundantly present in ascites, binds to its receptors highly expressed in ovarian cancer cells, activating MAPK, PI3K-AKT, and Rho GTPases [36]. These pathways induce angiogenic and inflammatory factors such as HIF1α, VEGF, IL-6, and IL-8, as well as proteases like MMPs and uPA, thereby driving angiogenesis, invasion, and growth factor signaling [36]. Ovarian cancer cells also increase cholesterol uptake through LDL receptor expression and LXR/SREBP2-mediated synthesis, which stiffens membranes, organizes pro-survival nanodomains, and upregulates drug efflux pumps including ABCG2 and MDR1 [37]. Moreover, prostaglandins−potent bioactive lipids enriched in ascites−create a pro-inflammatory milieu that activates cyclooxygenase, fueling pro-tumorigenic cascades such as PKA, PI3K-AKT, and Ras-ERK signaling [1].

Recent studies demonstrate that exosomal miRNAs from ovarian cancer ascites reprogram cancer cells. Notably, malignant ascites EVs display a distinct miRNA profile, including enrichment of miR-200a/b/c, miR-1246, and miR-1290, along with reduced levels of miR-100–5p, which collectively promote spheroid expansion, motility, and omental colonization [38]. In addition, miR-6780b-5p promotes EMT phenotypes in ovarian cancer cells, enhancing migration and metastasis, while miR-891–5p activates intracellular DNA repair pathways, enabling cancer cells to acquire chemoresistance and facilitating recurrence [39,40]. Beyond direct effects on tumor cells, exosomes derived from malignant ascites also reprogram peritoneal mesothelial cells into tumor-promoting phenotypes and contribute to the differentiation of cancer-associated fibroblasts and TAMs, thereby fostering a supportive microenvironment that sustains cancer cell proliferation, invasion, and immune evasion [1].

Iron in malignant ascites plays a dual role in ovarian cancer progression, highlighting the so-called “iron paradox” [41]. Proteomic profiling of cell-free ascites indicates that iron and iron-related proteins are elevated compared with cancer-free donor serum, meeting the high metabolic demands of cancer cells by supporting DNA synthesis, mitochondrial respiration, and anabolic growth; and ovarian tumor-initiating cells further sustain this supply by upregulating transferrin receptor 1 (TFR1, an iron importer) and downregulating ferroportin (FPN, an iron exporter), thereby ensuring continuous iron influx into metastatic cells [21,41]. Conversely, excess iron can trigger ferroptosis, an iron-dependent form of cell death, creating selective pressure for tumor cells to activate protective pathways [42]. To counteract this, ovarian cancer cells sequester labile iron through ferritin heavy and light chains (FTH1, FTL) and engage DNA repair programs via the POLQ/RAD51 axis, limiting genomic stress and enhancing chemoresistance [22]. At the same time, SCD1-mediated monounsaturated fatty acids (MUFA) production protects cancer cells from ferroptosis, an iron-dependent form of oxidative cell death [43]. Beyond intrinsic survival, iron metabolism also facilitates immune evasion by reducing ferroptotic damage that would otherwise release danger signals and by modulating macrophage and T cell function within ascites [41]. Thus, iron and its regulators function not merely as biomarkers but as active drivers of tumor adaptation, with their balance between promoting growth and restraining ferroptosis emerging as a central axis of ovarian cancer progression and therapeutic resistance.

Environmental components of ascites, including hypoxia, nutrient deprivation, and elevated IPP, reprogram ovarian cancer cell metabolism and promote tumor progression. In particular, fluid accumulation-induced increases in IPP stimulate remodeling of the peritoneal mesothelial cell surface and facilitates tunneling nanotube-mediated interactions [26]. Under compressive stress, these nanotubes transfer mitochondria from mesothelial cells to ovarian cancer cells, thereby supporting metabolic adaptation and fostering chemoresistance [26]. Elevated IPP also leads to tumor-associated collagen signatures, where straightened collagen fibers enhance cancer cell migration and metastasis by altering adhesion dynamics and reshaping the peritoneal microenvironment [26]. Collectively, these physical and metabolic changes induced by ascites contribute to ovarian cancer recurrence and dissemination.

## Immune dysfunction driven by intracellular stress

The immune system relies on intricate interactions among diverse immune cells to mount an effective defense against cancer. However, the extreme environment of malignant ascites−characterized by hypoxia, nutrient deprivation, and elevated levels of reactive oxygen species (ROS)−imposes substantial intracellular stress on immune cells, compromising their function and facilitating tumor immune evasion [44]. Hypoxia and limited nutrients in malignant ascitic milieu induce profound metabolic stress in immune cells: stabilized hypoxia-inducible factors (HIFs) reprogram cellular metabolism by upregulating glycolysis and suppressing oxidative phosphorylation, shifting energy production pathways, while competition for scarce glucose and amino acids further exacerbates these metabolic challenges, impairing glycolytic capacity and effector functions of CD8^+^ T cells [[44], [45], [46]]. In addition, amino acid scarcity suppresses mTOR signaling and global protein synthesis, exacerbating immune cell dysfunction [44]. The high levels of ROS damage cellular components, including lipids, proteins, and DNA [24]. This oxidative stress particularly affects mitochondria, the primary source and target of ROS, reducing ATP production and releasing pro-apoptotic signals that drive immune cell exhaustion [46].

Under this harsh ascitic microenvironment, immune cells activate stress-response programs and adjust their cellular behavior. Autophagy, for instance, helps mitigate oxidative stress by removing damaged mitochondria and limiting ROS through a process known as mitophagy [47]. In Tim-4^+^ TAMs or macrophages positive for complement receptor of the immunoglobulin superfamily in ovarian cancer patients, mitophagy is induced by the high level of arginase-1 and suppressed mTORC1, alleviating oxidative stress and maintaining mitochondrial fitness [47]. Consequently, autophagy inhibition in these TAMs results in their depletion, underscoring its critical role in the ascitic microenvironment [47]. Malignant ascites also directly skews macrophages: free arachidonic acid in ascites hinders STAT1 phosphorylation and disrupts receptor-JAK-STAT signaling, promoting CD163 and CD206 expression and M2-like polarization [48]. In addition, hypoxia-driven HIF signaling increases tumor exosome release, delivering miRNAs to TAMs and further reinforcing M2 polarization−an immune state linked to poor prognosis and reduced survival rates in ovarian cancer patients [49]. TAMs contribute to T cell exhaustion via engaging PD-1 receptor on T cells and secretion of immunosuppressive factors such as IL-10, TGF-β, and ROS, collectively blunting CD8^+^ tumor-infiltrating lymphocytes function [49].

Malignant ascites also inhibits NK cell activity, partly due to the presence of CA125 shed from ovarian tumors [1]. RNA sequencing data (GSE153713) reveal that NK cells exposed to ovarian cancer ascites, exhibit transcriptional downregulation of cytotoxic pathways, including type I IFN, activating receptors, and phosphatidylinositol signaling, which impairs their function [50]. CD3^−^CD56^+^CD16^+^ NK cells in ascites exhibit reduced CD16 expression, resulting in decreased proliferation, cytotoxicity, and cytokine production, despite being more enriched in ascites than in peripheral blood (11.0 % vs. 5.6 %) [51]. Recent lipidomics work shows that ovarian cancer ascites is lipid-replete and induces NK cell dysfunction through uptake of polar lipids, which remodel the NK cell lipidome [52]. Mechanistically, ascites-exposed NK cells upregulate lipid transporters such as SR-B1, accumulate specific phospholipids, lose neutral-lipid buffering capacity and secrete triacylglycerols, and disrupt plasma-membrane order, thereby blunting degranulation, granzyme/perforin expression, and cytotoxicity [52]. Blocking lipid uptake via SR-B1 restores NK cell activation, GLUT1 expression, membrane order, and cytotoxic function, establishing a direct metabolic-biophysical axis from ascitic lipids to NK suppression and revealing membrane/lipid-transport nodes as therapeutic targets [52].

Beyond metabolic stress, the endoplasmic reticulum (ER) stress response is another major mechanism for inducing immune dysfunction in malignant ovarian ascites [44]. Intracellular stress often leads to the accumulation of unfolded and misfolded proteins within the ER, activating the unfolded protein response (UPR) activation, mediated by three ER membrane-resident sensors, including Inositol-requiring enzyme 1α (IRE1 α), protein kinase RNA-like ER kinase (PERK), and activating transcription factor 6 (ATF6) [53]. While acutely cytoprotective−enhancing protein folding, attenuating protein synthesis, and promoting ER-associated degradation− prolonged and unresolved UPR activation fosters immune dysfunction and tumor-promoting inflammation, making it a critical mechanism underlying immune evasion in cancer [53]. In ovarian cancer, tumor-infiltrating dendritic cells (DCs) undergoing ER stress over-activate the IRE1α-X-box binding protein 1 (XBP1) pathway [54]. Ascites exposure drives ROS and intracellular 4-HNE-protein adducts in DCs, triggering lipid peroxidation and inducing ER stress; subsequent activation of XBP1 reprograms lipid metabolism by upregulating triglyceride biosynthesis and drives lipid-droplet accumulation, and impairs antigen processing and presentation, thereby diminishing CD8^+^ T cell cross-priming [54]. Similarly, high-grade serious ovarian cancer activates IRE1α in tumor-infiltrating neutrophils, which suppress T cell cytotoxicity; neutrophil-specific IRE1α deletion delays tumor growth and extends survival by enhancing T cell activities in vivo [55].

**Fig. 3:**
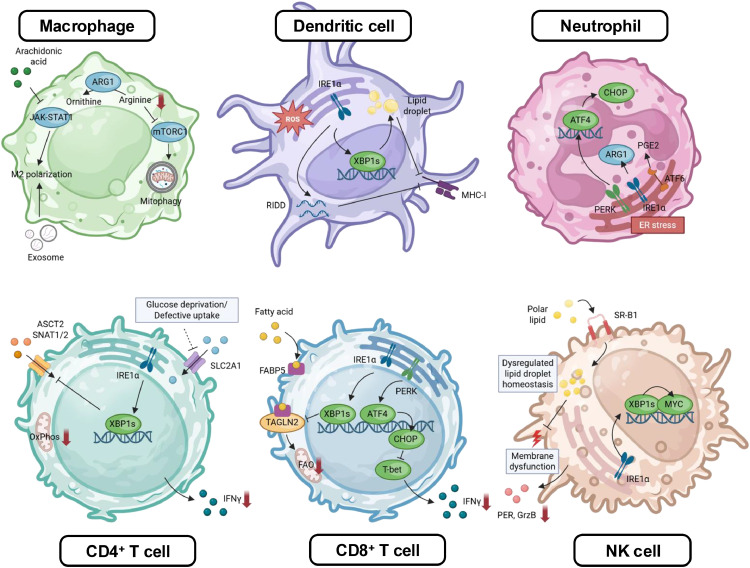
Immune dysfunction driven by intracellular stress. In ovarian ascites, hypoxia, altered nutrient uptake, dysregulated lipid species, and ROS trigger metabolic and ER stress in immune cells. Activation of the UPR branches−IRE1α- XBP1, PERK-ATF4-CHOP, and ATF6−further regulates metabolic and transcriptional programs, promoting immunosuppressive phenotypes and suppressing antitumor activity. Abbreviations: ARG1, arginase-1; ROS, reactive oxygen species; RIDD, regulated IRE1α dependent decay; OXPHOS, oxidative phosphorylation; FAO, fatty-acid oxidation; FABP5, fatty-acid–binding protein 5; PGE₂, prostaglandin E₂; TAGLN2, transgelin-2; SR-B1, scavenger receptor class B type 1. This image was created with BioRender.com.

T cells are likewise susceptible to ER-metabolic coupling. Glucose deprivation or reduced glucose transporter type 1 expression driven by tumor-derived factors in ascites, leads to defective N-linked glycosylation that triggers ER stress and further activates IRE1α-XBP1, leading to reduced glutamine uptake, impaired bioenergetics and mitochondrial respiration, ultimately resulting in T cell dysfunction [56]. Ovarian cancer ascites also provokes ER stress in CD8^+^ T cells that suppresses transgelin-2, disrupting cytoskeletal organization and fatty acid uptake via mis-localization of FABP5, thereby reducing fatty acid uptake, mitochondrial activity and cytotoxic capacity in CD8^+^ T cells; restoring transgelin-2 rescues lipid uptake, mitochondrial function, and killing [57]. Additionally, the ER stress-inducing transcription factor, CHOP, downregulates T-bet, which is a crucial regulator of CD8^+^ T cell cytotoxicity, through the PERK-ATF4 axis, further compromising CD8^+^ T cell cytotoxicity; elevated CHOP expression in CD8^+^ T cells is associated with poor outcomes and reduced anti-tumor function [58]. Collectively, malignant ascites imposes intertwined metabolic and ER stress pressures that reprogram TAMs, DCs, neutrophils, NK cells and T cells, dismantling anti-tumor immunity (Fig. 3).

## Clinical translation in ascites-predominant ovarian cancer

**Fig. 4:**
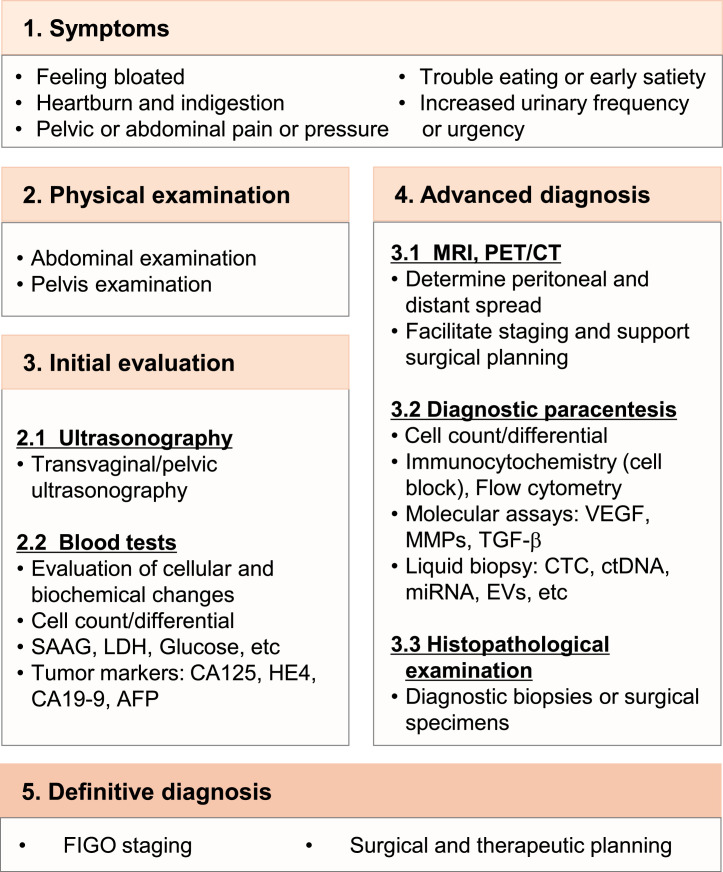
Diagnostic workflow for ovarian cancer patients with malignant ascites. The process begins with symptom assessment and physical examination, followed by initial evaluation with ultrasonography and blood tests. Advanced diagnosis incorporates crosssectional imaging, diagnostic paracentesis with cytology, immunocytochemistry, and molecular assays, liquid biopsy, as well as histopathological confirmation. Final integration establishes definitive diagnosis, FIGO staging, and guides surgical and therapeutic planning.

Malignant ascites represents one of the most debilitating complications in advanced ovarian cancer, arising from a complex interplay of vascular permeability, peritoneal tumor dissemination, metabolic adaptation, and immune suppression [59]. Recently, therapeutic development has expanded beyond palliative drainage toward mechanistic interventions that address the cellular and molecular underpinnings of ascites formation [59]. The following section delineates the detection and diagnostic methods (Fig. 4) and summarizes the major categories of therapeutic approaches, highlighting their molecular targets, biological rationale, expected benefits, and clinical trial status (Table 2).

**Table 2:** Therapeutic strategies and trial landscape controlling advanced ovarian cancer.

Categorization based on ascites formation process	Modality	Target (Cellular/Molecular)	Working mechanism	Ovarian cancer setting	ClinicalTrials. gov ID	Status
Vascular permeability/ influx control	Bevacizumab (± Chemotherapy)	VEGF-α	Neutralizes VEGF-α; Reduced microvascular permeability; Normalization window for drug delivery	First-line and recurrent high-risk OC	NCT00262847 NCT00483782	Completed [65,66]
	Aflibercept	VEGF-α/β/PIGF	Ligand trap; Reduced vascular leak; Prolongs paracentesis-free interval in malignant ascites	Recurrent, symptomatic malignant ascites including OC	NCT00396591	Completed [67]
	Pazopanib	VEGFR/PDGFR/c-KIT TKI	Multi-TKI; anti-angiogenesis and reduced leak	Maintenance after first-line therapy	NCT00866697	Completed [68]
	Nintedanib + carboplatin/paclitaxel	VEGFR/FGFR/PDGFR TKI	Multi-angiokinase blockade	First-line combination	NCT01015118	Completed [69]
	Cediranib Maleate+ Olaparib	VEGFR-1/2/3 TKI + PARP	Anti-angiogenic + DNA-repair blockade; potential synergy in hypoxic/stressful TME	Platinum-sensitive recurrent HGSOC	NCT01116648	Completed [70]
Locoregional control of peritoneal spread	HIPEC (Hyperthermic Intraperitoneal Chemotherapy)	Microscopic peritoneal tumor beds	Hyperthermic intraperitoneal chemotherapy at surgery	After NACT (interval debulking); selected relapses	NCT03772028	Recruiting/Active [72]
	PIPAC (Pressurized Intraperitoneal Aerosol Chemotherapy)	Peritoneal implants	Aerosolized, pressurized IP delivery; deeper distribution	Platinum-resistant / peritoneal carcinomatosis	NCT04329494	Recruiting/Active, feasibility and activity in OC cohorts [73]
Suppressive immune/ TME reprogramming	MK-4830 + pembrolizumab	ILT4/LILRB2 on myeloid cells	Myeloid checkpoint blockade; reprograms TAM/MDSC; restores T-cell priming	HGSOC, neoadjuvant platform	NCT05446870	Ongoing; ctDNA change specified as a key endpoint [75,96]
	IO-108 (± pembrolizumab)	ILT4/LILRB2 on myeloid cells	Myeloid reprogramming	Solid-tumor basket with OC cohort	NCT05054348	Ongoing phase 1/1b expansion [76]
	ZEN003694 + Talazoparib	BET + PARP	Suppress Notch3 and reduces tumor growth; reprogramming TAMs	Recurrent OC	NCT05071937	Recruiting (phase II)
	Evorpacept (ALX148) + Liposomal Doxorubicin+ Pembrolizumab	CD47 decoy blocker	Releases macrophage phagocytosis with low anemia risk	Platinum-resistant OC	NCT05467670	Ongoing phase II
	Magrolimab + Avelumab	CD47 mAb+PD-L1	Blocks “don’t-eat-me” signal; macrophage-mediated kill	OC cohort within phase 1b/2	NCT03558139	Completed early-phase cohort; safety/SD signals [78]
	INCB001158 (CB-1158) (± perbrolizumab; +chemotherapy)	Arginase 1/2 in myeloid cells and CAFs	Restores l-arginine; T-cell metabolic fitness; dampens MDSC function	Solid-tumor basket with OC cohort	NCT02903914 NCT03314935	Completed; safety/biologic activity; limited single-agent tumor shrinkage [80]
	OATD-02	Dual Arginase 1/2	Restores l-arginine; polyamine depletion, enhanced response to checkpoint blockade	Advanced/metastatic solid tumors	NCT05759923	Ongoing (phase I/II) [81]
	Pegcetacoplan (APL-2) (± pembrolizumab)	Complement C3	Inhibit complement-induced inflammation and suppressive immune axis	Recurrent OC	NCT04919629	Ongoing [82]
	E7777 (DD) + Pembrolizumab	Treg	Deplete Tregs	Recurrent, platinum resistant OC	NCT05200559	Recruiting [83]
	THEO-260	Cancer cells, CAFs	Oncolytic virus targeting cancer cells and CAFs; tumor lysis	Relapse, refractory OC	NCT06618235	Recruiting (phase I/IIa) [85]
	HCW9218	Bifunctional protein of TGF-β + IL-15	Sequester TGF-β and stimulate immune effector cells via IL-15; enhance anti-tumor immunity; overcome TME	Advanced solid tumors including recurrent OC cohort	NCT05322408	Active, not recruiting [86]
Stress and metabolic pathways to shape TME	HC-5404	PERK (UPR/ISR)	Inhibits PERK–eIF2α–ATF4 signaling; reduces stress-adapted survival; may restore T-cell function	Solid tumors	NCT04834778	Phase 1a completed [88]
	ORIN1001	IRE1α RNase/XBP1s	Dampens UPR-XBP1s programs that drive immune dysfunction and resistance	Solid-tumor basket with OC cohort	NCT05154201	Ongoing (phase I/II) [89]
	HC-7366 + Belzutifan	GCN2 activator + HIF2α blocker	Activating integrated stress response (ISR)	Advanced or metastatic RCC	NCT06234605	Ongoing early phase [90]
	Devimistat (CPI-613)	PDH/α-KGDH(TCA)	Disrupts TCA flux; chemo-sensitization of stress-adapted cells	Solid tumors with refractory OC	NCT05733000	On-going [91]
	Denifanstat (TVB-2640)	FASN	Blocks de novo lipogenesis; targets lipid-driven remodeling/ferroptosis tolerance	Solid tumors (OC-relevant biology)	NCT03032484 NCT03179904	Ongoing early phase in solid tumors [92]
Outflow and drainage control	Alfapump	Peritoneal → bladder	Continuous active drainage to bladder; reduces paracentesis need	Refractory malignant ascites with OC cohort	NCT03973866	Ongoing [94]
	Tunneled indwelling peritoneal catheter (e.g., PleurX)	Peritoneal cavity	Home-based intermittent drainage; symptom relief	Cancer-related malignant ascites	NCT02975726	Prospective/ observational programs active [95]

### Detection and diagnosis

According to the NCCN guidelines, common presenting features of ovarian cancer with ascites include abdominal bloating, indigestion, pelvic or abdominal pain or pressure, early satiety, and increased urinary frequency or urgency [60]. Clinical suspicion warrants abdominal and pelvic examination, followed by transvaginal or pelvic ultrasonography as the first-line test to confirm free fluid, estimate volume, and screen for adnexal or peritoneal masses [59]. Laboratory evaluation includes complete blood count, metabolic panel, LDH, glucose, and albumin for calculating the SAAG, which is typically <1.1 g/dL in peritoneal carcinomatosis [59]. Tumor markers such as CA125, HE4, CA19–9, and AFP support risk stratification and treatment monitoring, though they are not diagnostic on their own [61].

Advanced work-up combines cross-sectional imaging (contrast-enhanced CT or MRI, selectively FDG-PET/CT) to define peritoneal spread and surgical feasibility [59]. Ultrasound-guided diagnostic paracentesis is pivotal, with fluid submitted for cell count/differential, biochemical assays (albumin and total protein, ferritin, fibronectin, LDH, glucose, ADA), and cytology with cell-block preparation, supplemented by immunocytochemistry (PAX8, WT1, EpCAM, p53 patterning) to confirm Müllerian origin and exclude mimics, as well as flow cytometry for hematologic differentials [59]. Molecular adjuncts on supernatant or cell-block (VEGF, MMPs, TGF-β) can provide tumor genomic insight and inform trial eligibility [59]. Recent advances also highlight liquid biopsy approaches−including circulating tumor cells, ctDNA, cf-mRNA, and EV-miRNA profiling−as promising tools for early detection, monitoring progression, and anticipating drug resistance [11]. When cytology is negative, histopathologic confirmation through image-guided biopsy of peritoneal/omental implants, diagnostic laparoscopy, or surgical sampling at primary/interval debulking is required to establish histotype and enable genomic testing (e.g., BRCA1/2, HRD) [61,62]. Integration of clinical, imaging, cytologic/molecular, and histologic data allows accurate FIGO staging and guides personalized treatment, including cytoreductive strategies, neoadjuvant chemotherapy, along with maintenance with anti-angiogenic or PARP inhibitors [11]. Recent advances in deep learning models that extract and analyze patterns from medical images, as well as algorithms that integrate CA125 detection with PET/CT imaging for automated early diagnosis of ovarian cancer, are expected to enhance diagnostic accuracy [63,64].

### Therapeutic strategies and trial landscape

One of the principal mechanisms driving malignant ascites is VEGF-mediated vascular permeability, and accordingly a range of anti-angiogenic strategies have been investigated. Bevacizumab, a monoclonal antibody targeting VEGF-α, when added to first-line carboplatin-paclitaxel, improves progression-free survival (PFS), with the greatest benefit observed in high-risk patients, and reduces the frequency of paracentesis, thereby establishing maintenance bevacizumab as a standard therapeutic option (NCT00262847, NCT00483782) [65,66]. Aflibercept, a soluble decoy receptor for VEGF-α, VEGF-β and PIGF, effectively sequesters angiogenic ligands and prolongs paracentesis-free intervals in recurrent symptomatic malignant ascites (NCT00396591) [67]. Multi-target tyrosine kinase inhibitors (TKIs) have also been evaluated: pazopanib (VEGFR/PDGFR/c-KIT TKI) modestly improved PFS in the maintenance setting without an overall survival (OS) advantage (NCT00866697) [68], while nintedanib (VEGFR/FGFR/PDGFR TKI) improved PFS when combined with first-line chemotherapy but raised tolerability concerns (NCT01015118) [69]. In platinum-sensitive recurrent HGSOC, cediranib maleate, a pan-VEGFR TKI, demonstrated synergistic activity with the PARP inhibitor olaparib, leading to superior PFS compared with olaparib monotherapy (NCT01116648) [70]. Despite these advances, resistance to anti-VEGF therapy remains a major challenge. Mechanistic studies implicate IL-6/JAK/STAT3 hyperactivation as a compensatory pathway under VEGF blockade, promoting tumor survival and immunosuppressive remodeling [71]. Moreover, anti-VEGF-induced hypoxia fosters recruitment of MDSCs, further dampening anti-tumor immunity [71]. These insights provide a rationale for combinatorial approaches that pair VEGF blockade with inhibitors of IL-6, its receptor, or JAK kinases, aiming to mitigate resistance and enhance therapeutic efficacy [71].

Peritoneal implants represent a persistent source of malignant ascites, driving interest in locoregional strategies designed to eradicate microscopic tumor deposits and optimize intraperitoneal drug exposure [59]. Hyperthermic intraperitoneal chemotherapy (HIPEC) augments platinum cytotoxicity and tissue penetration, and when administered after interval debulking surgery, has been shown in randomized trials to improve recurrence-free survival (NCT03772028) [72]. More recently, pressurized intraperitoneal aerosol chemotherapy (PIPAC) has emerged as a minimally invasive approach, in which pressurization facilitates homogeneous drug distribution across the peritoneal surface [73]. Early clinical studies demonstrate its safety and feasibility, particularly in patients with platinum-resistant peritoneal carcinomatosis (NCT04329494) [73].

Malignant ascites is enriched in immunosuppressive myeloid cells, Tregs, and CAFs, all of which collaborate to establish a profoundly inhibitory milieu for anti-tumor immunity [1]. Emerging strategies seek to reprogram this landscape, dismantling multiple layers of suppression to restore effective innate and adaptive responses. Inhibitory receptors such as ILT4/LILRB2 rewire myeloid cells toward tolerogenic programming [74]. Blockades including MK-4830 and IO-108 are under evaluation (NCT05446870, NCT05054348), with the goal of restoring antigen presentation and T cell priming while curbing MDSCs/TAMs-mediated immunosuppression [75,76]. In parallel, BET inhibition with ZEN003694 suppresses Notch3 and tumor growth while reprogramming macrophages; its combination with the PARP inhibitor talazoparib is being investigated in recurrent ovarian cancer (NCT05071937). Ovarian cancer cells exploit CD47 to deliver a “don’t eat me” signal, shielding themselves from phagocytosis [77]. Inhibitors such as ALX148 and Magrolimab disrupt this axis, enhancing macrophage-mediated clearance and innate immune surveillance [77]. Early-phase trials in ovarian cancer cohorts report safety and signs of activity when combined with chemotherapy or checkpoint blockade (NCT05467670, NCT03558139) [78]. Other approaches address metabolic suppression. l-arginine depletion by myeloid cells and CAFs paralyzes T cell metabolism; arginase inhibitors are therefore expected to restore arginine availability and effector function [79]. The oral agent INCB001158 showed tolerability and on-target pharmacodynamic effects but limited monotherapy activity, underscoring the need for combinations (NCT02903914, NCT03314935) [80]. In addition, the dual arginase-1/2 inhibitor OATD-02 has entered a phase I clinical trial (NCT05759923) [81]. Complement overactivation also promotes inflammatory immunosuppression; inhibition with pegcetacoplan (APL-2) targets C3 to reduce pro-tumor inflammation and rebalances the immune axis toward anti-tumor (NCT04919629) [82].

Tregs are enriched in ascites and represent a major barrier to checkpoint efficacy. The IL-2/diphtheria toxin fusion protein E7777 (denileukin diftitox, DD) depletes Tregs; while single-agent activity was modest in Phase II trials, combinations with pegylated IFN-α2a have shown enhanced immune and clinical benefits, and E7777 is currently being evaluated in combination with Pembrolizumab (anti-PD-1) for recurrent or metastatic ovarian cancer (NCT05200559) [83]. CAF-directed approaches are also advancing; THEO-260, an oncolytic virus engineered to target tumor and stromal antigens (EpCAM, EGFR, PD-L1, CA125, FAP), induces immunogenic cell death and effector T cell responses, with encouraging activity in preclinical ovarian cancer models [84]. A Phase I/IIa trial in refractory disease is ongoing (NCT06618235) [85]. Finally, cytokines prevalent in ascites emerge as therapeutic targets. HCW9218, a bifunctional protein complex that suppresses TGF-β while stimulating IL-15 signaling, demonstrated potent immune activation in early-phase studies (NCT05322408), including marked expansion of NK cells [86]. Taken together, combining myeloid checkpoint blockade, metabolic rescue, Treg/CAF depletion, and cytokine modulation offers a coherent strategy to dismantle the ascitic immune shield and enhance responses to chemo- and immunotherapies in ovarian cancer.

Single cell RNA-seq revealed that chemotherapy in ovarian cancer enriches a stress-associated cell state, that drives chemoresistance, worsens prognosis, and is sustained by a paracrine loop with inflammatory CAFs (iCAFs) [12]. This state appears to be largely non-genetic and adaptive, underscoring the therapeutic potential of targeting stress and metabolic pathways that shape the hostile TME and suppress antitumor immunity [12]. Several agents are currently under development to intervene in these pathways, each with distinct molecular targets and mechanistic rationale. Under ER stress, PERK phosphorylates eIF2α, transiently shutting down global translation while selectively inducing ATF4/CHOP, which promotes survival, angiogenesis, and therapy tolerance, while restrain anti-tumor CD8^+^ T cell activity [87]. HC-5404 is a potent, selective PERK inhibitor which attenuates the UPR and integrated stress response (ISR) and a phase 1a trial in solid tumors has been completed (NCT04834778), providing initial safety data [88]. Mechanistically, PERK inhibitor removes the ISR survival crutch and is expected to improve chemosensitivity and relieve T cell suppression driven by CHOP [88]. The IRE1α RNase/XBP1s augments lipid metabolism and secretory capacity and, in ovarian cancer, contributes to immune dysfunction and drug resistance [87]. ORIN1001, selectively inhibits IRE1α RNase preventing XBP1s generation; it is being evaluated in a solid-tumor basket trial including an ovarian cancer cohort (NCT05154201), with an ongoing phase 1/2 study [89].

Beyond classical UPR signaling, combined GCN2 activator (HC-7366) that enforces amino acid-stress signaling, limiting anabolic drive in starved tumors and HIF2α inhibitor (Belzutifan) that blocks hypoxia-driven transcription targets dual stress adaptations, with a phase 1b study ongoing (NCT062334605), particularly relevant in advanced or metastatic renal cell carcinoma, where an early-phase trial is ongoing [90]. Moreover, metabolic vulnerabilities of stress-adapted cells are also being explored. Devimistat (CPI-613) targets pyruvate dehydrogenase (PDH) and α-ketoglutarate dehydrogenase (α-KGDH) within the tricarboxylic acid (TCA) cycle, disrupting central metabolic fluxes and sensitizing stress-adapted ovarian cancer cells to chemotherapy [91]. This agent is currently under investigation in multi-cohort including refractory ovarian cancer (NCT05733000) [91]. Stress-adapted and ascites-exposed tumors elevate FASN for de novo lipogenesis, supporting membrane biogenesis, signaling-lipid pools, and ferroptosis resistance. Denifanstat (TVB-2640) inhibits FASN, and clinical data show it is combinable with bevacizumab and biologically active in solid tumors (NCT03032484), with additional synergy seen preclinically (NCT03179904) [92]. Mechanistically, FASN blockade is expected to limit the lipogenic remodeling that sustains immune evasion and therapy tolerance within lipid-rich peritoneal niches [93]. Together, these agents aim to disrupt stress and metabolic adaptations that sustain chemoresistant and immunosuppressive TMEs; by targeting both tumor-intrinsic and stromal-driven pathways, such strategies hold promise for overcoming resistance and improving patient outcomes in ovarian cancer.

Despite progress in mechanistic therapies, continuous accumulation of malignant ascites remains a major cause of morbidity, necessitating supportive interventions to improve quality of life. Device-based solutions provide durable palliation: the alfapump system continuously transfers ascites to the bladder, reducing paracentesis frequency (NCT03973866), while tunneled indwelling peritoneal catheters (e.g., PleurX) enable at-home intermittent drainage, improving patient autonomy (NCT02975726) [94,95]. These approaches do not modify tumor biology directly but address the symptomatic burden of ascites, highlighting the need for integrated management strategies that combine biological therapies to suppress ascites formation with palliative tools to control fluid accumulation.

## Conclusion

Malignant ascites is not a passive effusion but a biologically active compartment that sustains peritoneal persistence, invasion, and treatment tolerance in advanced ovarian cancer. Convergent intracellular stress programs−most notably UPR-metabolic coupling−reconfigure antigen-presenting cells and cytotoxic lymphocytes while reinforcing tumor lipid dependence and ferroptosis avoidance. Clinically, an imaging-guided diagnostic pathway with selective use of paracentesis/cell-block, liquid biopsy, and careful distinction from non-malignant ascites streamline decision-making. Therapeutically, multi-pronged strategies that combine VEGF-axis control with immune reprogramming and stress/metabolic inhibitors provide a coherent framework to dismantle the ascitic niche. Future trials should prospectively incorporate ascites-specific biomarkers (EV-miRNAs, lipidomics, cfDNA), test mechanism-guided combinations, and prioritize patient-centered endpoints (paracentesis-free intervals, symptom relief, and quality of life). Reframing ascites as a targetable microenvironment is essential to achieving durable disease control in ovarian cancer.

## Funding sources

This research was supported by the 10.13039/501100003645National Cancer Center, Korea (NCC-19112605), 10.13039/501100003725National Research Foundation of Korea (NRF) funded by the Korea Government (MSIT) (RS-2023–00213292, and RS-2024–00405650) (M.S.).

## CRediT authorship contribution statement

**Kyung Hyun Boo:** Writing – review & editing, Writing – original draft, Methodology, Conceptualization. **Gaeun Lee:** Writing – review & editing, Writing – original draft, Methodology, Conceptualization. **Minkyung Song:** Writing – review & editing, Writing – original draft, Supervision, Project administration, Funding acquisition, Conceptualization.

## Declaration of competing interest

The authors declare that they have no known competing financial interests or personal relationships that could have appeared to influence the work reported in this paper.
